# NDP‐*β*‐d‐*manno*‐heptoses are small molecule agonists sensed by the vertebrates to discriminate organisms of different kingdoms

**DOI:** 10.1002/ctm2.70103

**Published:** 2024-11-18

**Authors:** Yue Tang, Zijian Zhong, Huijin Mao, Yihua Chen

**Affiliations:** ^1^ State Key Laboratory of Microbial Resources Institute of Microbiology Chinese Academy of Sciences Beijing China; ^2^ College of Life Sciences University of Chinese Academy of Sciences Beijing China

**Keywords:** across kingdom, ALPK1, HBE, innate immune, NDP‐*β‐* d‐*manno*‐heptose, small molecule agonist

1

The mammalian innate immune system engages germline‐encoded pattern recognition receptors (PRRs) to sense the agonists from invading organisms to discriminate ‘self’ and ‘nonself’.^1^ Those agonists are usually large molecular signatures termed pathogen‐associated molecular patterns (PAMPs) or microbe‐associated molecular patterns (MAMPs), such as bacterial lipopolysaccharide (LPS) and fungal *β‐*glucan recognized by toll‐like receptor 4 or dectin‐1, respectively.^2^, ^3^ Notably, several recent works revealed that, besides the molecular patterns, specific small molecules can also act as agonists that elicit innate immune responses efficiently.^4^ Several *β‐* d‐*manno*‐heptose metabolites involved in the biosynthesis of LPSs have been identified as small molecule agonists that can be recognized by host alpha‐protein kinase 1 (ALPK1), with ADP‐d‐*glycero*‐*β‐* d‐*manno*‐heptose (ADP‐heptose) and its C‐6′′ epimer as the most potent ones.^5^ Upon binding to ADP‐heptose, ALPK1 will undergo conformational changes to phosphorylate TRAF‐interacting protein with forkhead‐associated domain (TIFA), and then triggers the activation of Nuclear factor kappa B (NF‐κB) and inflammation.

ADP‐heptose is synthesized from d‐sedoheptulose 7‐phosphate (S7P) via a four‐step relay catalyzed by NDP‐heptose biosynthetic enzymes (HBEs) with isomerase, kinase, phosphatase, and nucleotidyltransferase activities (Figure 1). Three types of HBEs with nucleotidyltransferase (HENase) activities were identified, including monodomain nucleotidyltransferase, didomain kinase/nucleotidyltransferase, and tridomain isomerase/kinase/nucleotidyltransferase.^6^ Before our work, knowledge of HBEs is limited to bacteria. We expanded the understanding of HBEs repertoire beyond the territory of bacteria to viruses, archaea, and eukaryotes.^7^ Enzymatic characterization of HBEs from different kingdoms verified that all of them could synthesize ADP‐heptose and some HENases could also recognize CTP and UTP to generate two new heptose metabolites, CDP‐d‐*glycero*‐*β‐* d‐*manno*‐heptose (CDP‐heptose) and UDP‐d‐*glycero*‐*β‐* d‐*manno*‐heptose (UDP‐heptose). Systematic evaluation of the NTP substrate scopes of HENases identified a conserved (F/L)XXG**R**STT motif (STT_R5_) as a hallmark of HENases with high NTP substrate promiscuity (Figure 1). The fifth arginine residue of the STT_R5_ motif may stabilize NTP in a reactive conformation by contributing cation‐π interaction with its nucleotide base and hydrogen bonds with its phosphate groups, thereby enabling the HENases to take different NTPs to produce not only ADP‐heptose but also CDP‐ and/or UDP‐heptoses. STT_R5_ could be found in all three types of HENases occurring in bacteria, archaea, and eukaryotes, suggesting that ADP‐, CDP‐. and UDP‐heptoses could be synthesized by a variety of organisms. The cellular levels of different NDP‐heptoses were detected in two representative pathogenic *Burkholderia* strains, revealing that all of the three NDP‐heptoses were accumulated to considerable amounts.^7^

**FIGURE 1:**
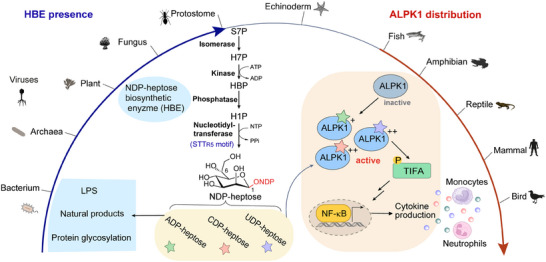
NDP‐heptoses are cross‐kingdom small molecule agonists of alpha‐protein kinase 1 (ALPK1)‐dependent innate immune responses. NDP‐heptose is synthesized from S7P via a four‐step relay catalyzed by NDP‐heptose biosynthetic enzymes (HBEs) with isomerase, kinase, phosphatase and nucleotidyltransferase activities. A conserved STT_R5_ motif was identified as a hallmark of the heptose nucleotidyltransferase that can synthesize not only ADP‐heptose but also CDP‐ and UDP‐heptoses. NDP‐heptoses can trigger ALPK1‐dependent innate immune responses via the Nuclear factor kappa B (NF‐κB) signalling cascade in mammalian cells, which eventually leads to the release of inflammatory cytokines and the recruitment of immune cells. HBEs are widely distributed in bacteria, archaea, viruses, plants, and protostomes (indicated with a blue arrow). ALPK1 homologs are present only in vertebrates (indicated with red arrow). ADP‐heptose participates in protein glycosylation and the biosynthesis of LPSs and natural products in bacteria. The physiological roles of the CDP‐ and UDP‐heptoses remain elusive. H7P, d‐*glycero*‐d‐*manno*‐heptose 7‐P; HBP, d‐*glycero*‐*β‐* d‐*manno*‐heptose 1,7‐PP; H1P, d‐*glycero*‐*β‐* d‐*manno*‐heptose 1‐P.

A comparison of the crystal structures of ALPK1‐CDP‐heptose and ALPK1‐ADP‐heptose complexes with the predicted structure of the ALPK1‐UDP‐heptose complex revealed a common binding pattern of these molecules. Not surprisingly, CDP‐ and UDP‐heptoses are also potent agonists that can activate the kinase activity of ALPK1 and drive it to phosphorylate TIFA with similar efficiencies as ADP‐heptose in vitro. While, tests in human and mouse cells showed that CDP‐ or UDP‐heptose can trigger much stronger ALPK1‐dependent innate immune responses than ADP‐heptose, which was also observed in the in vivo assays of mice. Further investigation showed that similar performances were observed when electroporation of ADP‐, CDP‐, or UDP‐heptose into 293 T cells, indicating a potential difference in the delivery efficiencies of the three metabolites into the host cells.^7^ The results raised interesting questions as that, for the ALPK1‐mediated immune responses elicited by organisms like *Burkholderia*, which of the three NDP‐heptoses is the most important agonist? Can we modulate the host's innate immunity by controlling the production of different NDP heptoses?

Analysis of the distribution of ALPK1 and TIFA homologs revealed a limited occurrence in vertebrates (Figure 1). In contrast, HBEs are widely distributed in bacteria, archaea, viruses, and some simple eukaryotes belonging to Protostomia, which implies that, after deuterostomes lost the ability to synthesize NDP‐heptose, some vertebrates evolved a signalling pathway using ALPK1 as the receptor to discriminate ‘themselves’ from numerous NDP‐heptose producers. ALPK1s from fishes, amphibians, birds, and mammals exhibited comparable efficiencies in activating the ALPK1‐TIFA‐NF‐*κ*B signalling cascade, indicating this immunity axis is quite conservative. Unlike PAMPs or MAMPs that are “molecular patterns” specific to certain groups of microorganisms, small molecule agonists like NDP‐heptose can be produced by organisms from different kingdoms, including microorganisms, plants (Dinophytes), and animals (Arthropoda, Mollusca, etc.), which enables the host to efficiently sense varied invaders via a common innate immunity receptor. The evolutionary relationship between HBEs and ALPK1s may offer a fresh perspective on how to search for other small‐molecule immune agonists and their PRRs.

The widespread occurrence of HBEs indicated that heptoses may play more biological roles than what we have known to date, especially in their producers. Actually, there are only limited studies to show that *manno*‐heptose can participate in the installation of cell wall components (e.g. LPSs and capsular polysaccharides), the post‐translational heptosylation of certain proteins, and the biosynthesis of natural products (e.g., septacidin) in bacteria.^8^, ^9^, ^10^ It was suggested that archaea *Methanococcus maripaludis* S2 possesses the ability to synthesize ADP‐heptose, but the reason why archaea tend to produce *manno*‐heptose remains elusive. The same questions are also unsolved in the HBE‐containing plants, animals, and viruses. Moreover, in addition to ADP‐heptose, a lot of the HBEs are capable of synthesizing CDP‐ and UDP‐heptoses, while few works have been done to understand the physiological roles of the newly discovered NDP‐heptoses. Analysis of the conserved genes adjacent to the HBE‐encoding genes may offer some hints about their physiological functions in bacteria, which tend to cluster functional related genes together. Knowledge from bacteria may provide some clues for tracking the roles of *manno*‐heptose in the other kingdoms.

Furthermore, considering the large amounts and significant diversity of HBEs, this group of enzymes deserve to be investigated deeply. There may be other *manno*‐heptose metabolites yet to be discovered. Besides, HBEs could serve as attractive drug targets for the treatment of notorious Gram‐negative bacterial pathogens that are resistant to antibiotics.

## AUTHOR CONTRIBUTIONS

All authors have contributed to writing the manuscript and have approved the final manuscript.

## CONFLICT OF INTEREST STATEMENT

The authors declare no conflict of interest.

## ETHICS STATEMENT

Not applicable.

## PATIENT CONSENT STATEMENT

Not applicable.
